# Value of Circulating Cytokine Profiling During Submaximal Exercise Testing in Myalgic Encephalomyelitis/Chronic Fatigue Syndrome

**DOI:** 10.1038/s41598-018-20941-w

**Published:** 2018-02-09

**Authors:** Kegan J. Moneghetti, Mehdi Skhiri, Kévin Contrepois, Yukari Kobayashi, Holden Maecker, Mark Davis, Michael Snyder, Francois Haddad, Jose G. Montoya

**Affiliations:** 1 Stanford Cardiovascular Institute, Stanford University, Stanford, CA USA; 2 Division of Cardiovascular Medicine, Stanford University School of Medicine, Stanford, CA USA; 3 Department of Medicine, St Vincent’s Hospital, University of Melbourne, Melbourne, Australia; 4 General Medical Disciplines, Department of Medicine, Stanford University School of Medicine, Stanford, CA USA; 5 Human Immune Monitoring Center, Institute for Immunity, Transplantation and Infection, Stanford University School of Medicine, Stanford, CA USA; 6 Division of Infectious Diseases, Stanford University School of Medicine, Stanford, CA USA; 7 Palo Alto Medical Foundation Toxoplasma Serology Laboratory, Palo Alto, CA USA

## Abstract

Myalgic Encephalomyelitis or Chronic Fatigue Syndrome (ME/CFS) is a heterogeneous syndrome in which patients often experience severe fatigue and malaise following exertion. Immune and cardiovascular dysfunction have been postulated to play a role in the pathophysiology. We therefore, examined whether cytokine profiling or cardiovascular testing following exercise would differentiate patients with ME/CFS. Twenty-four ME/CFS patients were matched to 24 sedentary controls and underwent cardiovascular and circulating immune profiling. Cardiovascular analysis included echocardiography, cardiopulmonary exercise and endothelial function testing. Cytokine and growth factor profiles were analyzed using a 51-plex Luminex bead kit at baseline and 18 hours following exercise. Cardiac structure and exercise capacity were similar between groups. Sparse partial least square discriminant analyses of cytokine profiles 18 hours post exercise offered the most reliable discrimination between ME/CFS and controls (κ = 0.62(0.34,0.84)). The most discriminatory cytokines post exercise were CD40L, platelet activator inhibitor, interleukin 1-β, interferon-α and CXCL1. In conclusion, cytokine profiling following exercise may help differentiate patients with ME/CFS from sedentary controls.

## Introduction

Myalgic encephalomyelitis also known as chronic fatigue syndrome (ME/CFS) is a complex and debilitating syndrome of unknown etiology affecting more than one million Americans and several millions of individuals worldwide^1,2^. ME/CFS is characterized by persistent or relapsing unexplained fatigue of at least 6 months duration that is not alleviated by rest and results in a substantial reduction of previous levels of occupational, educational, social, and personal activities. Patients with ME/CFS often experience post exertional malaise, which further limits exercise activity. The absence of a reliable diagnostic laboratory test or biomarker for ME/CFS presents a significant problem for patients, treating clinicians and the research community.

While the exact mechanisms underlying ME/CFS are not yet well define, previous studies have suggested contribution from cardiac or immune dysfunction. Early reports from small studies suggested that patients with ME/CFS may have small cardiac dimensions, decreased circumferential myocardial strain or increased vascular stiffness^3,4^. More recently, altered immune responses and cytokine profiles were observed in many patients with ME/CFS; this however, has not been consistently found by others^5–7^. A multicenter cross-sectional study of 298 patient with ME/CFS by Hornig *et al*. reported a distinct cytokine inflammatory signature associated with early versus late syndrome^8^. The study suggested a specific immune profile early in the course of ME/CFS, which may have implications for diagnosis and the timing of intervention. For example, reduced levels of CD40 ligand (CD40L) were seen in short duration disease and contrasting cytokines profiles were observed when compared to longer duration disease. Further work has recently highlighted the ability of select cytokines, in particularly interleukin (IL)-5, IL-6, Plasminogen activator inhibitor (PAI-1) and Granulocyte colony-stimulating factor (GCSF) to discriminate between those patients with typical and atypical symptoms in ME/CFS^9^.

In this study, we first sought to determine whether patients with ME/CFS have altered ventricular mechanics or increase myocardial stiffness when compared to well matched sedentary controls. We further sought to determine whether symptom limited exercise highlights differences in cytokine profiles between ME/CFS and sedentary controls. Patients with ME/CFS are known to have significant post exertional fatigue and changes in cytokine profiles following exercise may be accentuated. As a sub-objective, we also sought to describe using network analysis, changes that occur in cytokines and growth factors 18 hours following symptom limited exercise in both sedentary controls and patients with ME/CFS.

## Results

### Population

We screened a total of 84 individuals to identify 24 patients with ME/CFS as well as 24 matched healthy sedentary volunteers. We initially randomly selected 41 patients with ME/CFS of which 17 were excluded for the following reasons: six patients reported inability to exercise, four patients reported the inability to complete an exercise study, two patients had evidence of subclinical atherosclerosis (carotid plaque), two patients were on anti-inflammatory medication, and three patients chose not to participate. We screened 43 healthy volunteers; 14 were excluded because they had a high level of physical activity based on the International Physical Activity Questionnaire (IPAQ), one patient had a subclinical severe carotid stenosis, one patient had evidence of subclinical systolic ventricular dysfunction, two were on anti-inflammatory medication and one subject chose not to participate after discussion of the study protocol.

**Figure 1:**
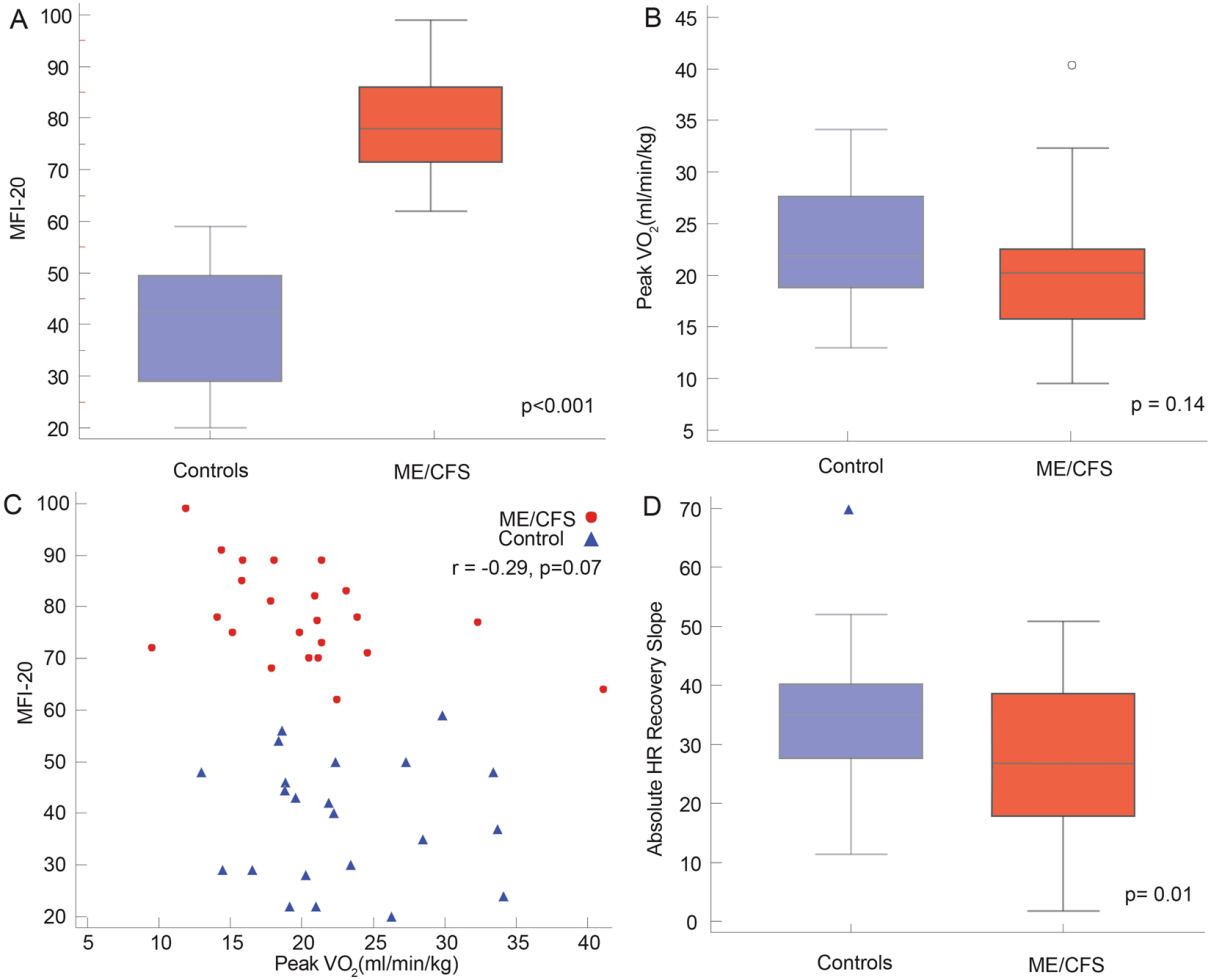
Clinical demographics of Controls and ME/CFS. There was a significant difference in MFI-20 between controls and participants with ME/CFS (**A**). Groups were similar with regard to maximal oxygen consumption (peak VO_2_) (**B**), however, MFI-20 did not correlate to peak VO_2_ (**C**). There was a reduction in absolute heart rate recovery slope in patients with ME/CFS when compared to controls (**D**).

The majority of patients with ME/CFS were female **(**Table 1**)**, with a median symptom duration of 9 years [ranging from 2–26]. The standardized fatigue questionnaire (MFI-20) score was significantly higher in patients with ME/CFS when compared to matched controls **(**Fig. 1A**)**. There was no significant difference in systolic blood pressure, heart rate, fasting glucose, lipid profile, high sensitivity C-reactive protein or thyroid stimulating hormone between groups.

**Table 1:** Comparison of the study groups.

Characteristics	CONTROLS (n = 24)	ME/CFS (n = 24)	*p*
Age (years)	46.1 ± 10.7	46.3 ± 10.9	0.92
Female	19 (79%)	19 (79%)	1
Caucasian Race	24	24	1
BMI (kg/m^2^)	25.5 ± 2.6	23.8 ± 3.5	0.03
BSA (m^2^)	1.85 ± 0.17	1.77 ± 0.20	0.03
MFI-20	40 ± 12	79 ± 9	<0.01
sBP (mmHg)	119 ± 12	118 ± 8	0.81
Heart rate (bpm)	64.5 ± 10.8	66.3 ± 11.0	0.52
***Biochemistry***			
Sodium (mmol/L)	143.8 ± 2.5	144.0 ± 2.2	0.78
Potassium (mmol/L)	4.36 ± 0.40	4.40 ± 0.42	0.72
HCO_2_ (mmol/L)	22.4 ± 1.9	23.0 ± 2.0	0.35
Chloride (mmol/L	104.1 ± 1.8	104.0 ± 2.1	0.98
Urea (mmol/L)	12.7 ± 4.1	12.4 ± 5.2	0.80
Creatinine (µmol/L)	76 ± 15	75 ± 12	0.87
Calcium (mgl/dL)	9.47 ± 0.34	9.45 ± 0.35	0.91
Fasting Glucose (mmol/L)	4.8 ± 0.4	4.9 ± 0.6	0.40
TC/HDL	3.5 ± 0.9	3.6 ± 1.0	0.75
TSH (mIU/L)	1.9 ± 1.3	1.8 ± 1.8	0.75
hs-CRP (mg/L)	2.7 ± 3.6	1.2 ± 1.1	0.10

BMI = body mass index; BSA = body surface area; ME/CFS = Chronic Fatigue Syndrome; hs-CRP – highly sensitive CRP, MFI – Multi dimensional fatigue inventory; SED = sedentary; sBP = systolic blood pressure; TSH – thyroid stimulating hormone; TC – Total cholesterol; HDL – High density lipoprotein.

### Cardiovascular phenotypes

There were no significant differences in left ventricular, right ventricular or vascular parameters between the groups (Table 2). Of importance scaled cardiac dimensions, left ventricular mass to volume ratio and left ventricular longitudinal strain did not differ between groups. Regarding assessment of endothelial function, the reactive hyperemia index (RHI) was similar in both groups at rest (2.21 ± 0.71 vs 2.44 ± 0.67, p = 0.07).

**Table 2:** Ultrasound Parameters.

Characteristics	CONTROLS (n = 24)	ME/CFS (n = 24)	*p*
**LV Parameters**			
LVEDD (mm)	48 ± 5	47 ± 4	0.06
LVEDVI (mL/m^2^)	58 ± 9	57 ± 11	0.31
LVMI (g/m^2^)	58 ± 10	57 ± 8	0.62
RWT	0.30 ± 0.03	0.30 ± 0.03	0.12
Mass:Volume	1.00 ± 0.10	1.01 ± 0.08	0.51
LVEF (%)	65 ± 6	64 ± 6	0.39
LVGLS (%)	−19.6 ± 1.5	−19.9 ± 2.1	0.36
LV E/e′	6.2 ± 2.2	5.7 ± 1.6	0.11
LAVI (mL/m^2^)	22 ± 5	20 ± 7	0.05
**RV Parameters**			
RVFAC (%)	49 ± 10	48 ± 8	0.87
TAPSE (mm)	2.5 ± 0.4	2.4 ± 0.4	0.21
RVSP (mmHg)	23 ± 4	22 ± 4	0.49
RAVI (mL/m^2^)	18 ± 5	17 ± 6	0.36
**Vascular parameters**			
cIMT (mm)	0.52 ± 0.13	0.52 ± 0.11	0.85
PWV (m/s)	6.7 ± 1.4	6.3 ± 1.2	0.22

ME/CFS = Chronic Fatigue Syndrome, LV = Left ventricle; LVEDD = Left ventricular end-diastolic diameter; LVEDVI = Left ventricle end-diastolic volume index; LAVI = Left atrium volume index; LVMI = Left ventricular mass index; RWT = Relative wall thickness; LVEF = Left ventricular ejection fraction; LVGLS = Left ventricular global longitudinal strain; PP = Pulse pressure; RV = Right ventricle, RVFAC = Right ventricular fractional area change, RSVP = Right ventricle systolic pressure, RAVI = Right atrial volume index, PWV = Pulsed wave velocity, SED = Sedentary, SVI = Stroke volume index, TAPSE = Tricuspid Annular Plane Systolic Excursion.

### Exercise parameters between both groups

All patients successfully completed a symptom limited one–day exercise protocol with no adverse events. The average peak respiratory exchange ratio was 1.14 ± 0.12 in patients with ME/CFS and 1.18 ± 0.10 in sedentary controls (p = 0.22). Although there was a clear separation between groups using MFI-20 questionnaire, there was no difference in the two groups in maximal heart rate achieved (147 ± 16 vs. 151 ± 16 bpm, p = 0.43), VE/VCO_2_ (25 ± 4 vs. 26 ± 5, p = 0.55) or peak VO_2_ (28.6 ± 6.7 vs. 29.7 ± 8.3 mL/kg/min, p = 0.23) **(**Fig. 1B**)**. There was no significant correlation between fatigue as measured by MFI-20 questionnaire and exercise performance as define by peak VO_2._ **(**Fig. 1C). There was no difference in RHI during recovery (2.40 ± 0.71 vs 2.12 ± 1.12, p = 0.14) or 18 hours post exercise (2.40 ± 0.70 vs 2.42 ± 0.68, p = 0.92). There was, however, a reduction in heart rate recovery slope in patients with ME/CFS when compared to controls (−26.8 ± 12.4 vs. −35.8 ± 17.4, p = 0.01) **(**Fig. 1D).

### Dynamic changes in cytokine markers following exercise (18 hours)

**Figure 2:**
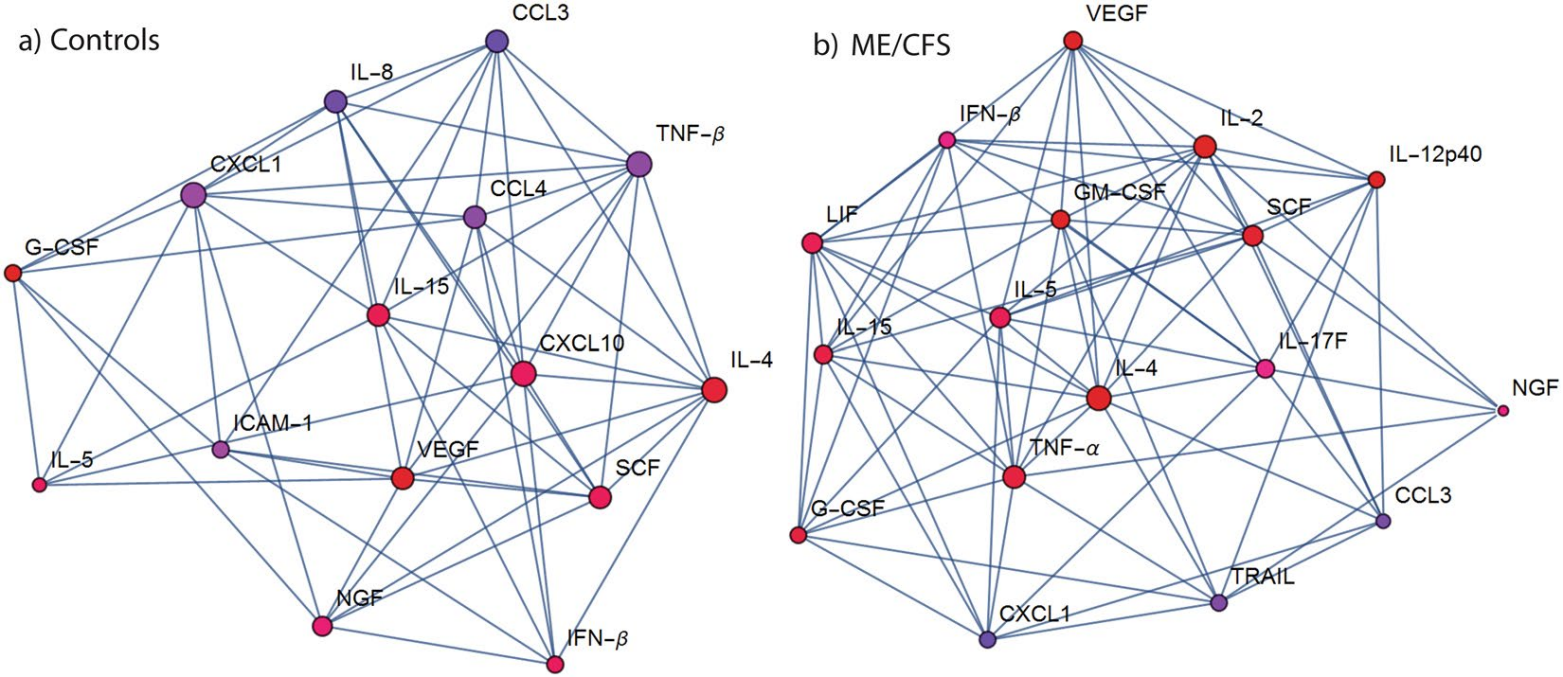
Network of the change in cytokines with exercise. Vertex colors are dark blue for strongly negative average delta to dark red for strongly positive average delta. Degree of centrality of cytokines is represented by the diameter of its vertex. The diameter of a cytokine’s vertex is proportional to its quantity of direct direct connections (blue lines) with other cytokines. In (**A**) Controls: IL-5, TNF-alpha and IL-2 are richly connected while in (**B**) ME/CFS: CXCL10, VEGF and IL-15 are richly interconnected. IL-4 appeared to play a similar role in both networks.

Following exercise, of the 51 cytokines and growth factors measured, 10 significantly changed after adjustment for multiple comparisons in both groups (8 increased and 2 decreased) **(**Table 3). A further seven only change in controls (IL-2, IL-12p40, IL-17F, LIF, TNF-α and GM-CSF) and five only in those with ME/CFS (CXCL10, IL-8, CCL4, TNF-β and ICAM-1). The dynamic change in cytokines were strongly associated to each other as highlighted by the network map **(**Fig. 2**)**. CXCL10, vascular endothelial growth factor (VEGF) and IL-15 were highly connected with other cytokines in the ME/CFS network. IL-5, TNF- α and IL-2 were richly connected in the control network. Leukemia inhibitory factor (LIF) also contributed to the control network, however not in participants with ME/CFS. While IL-4 appeared to be central to both networks, connections to IL-4 differed between case and control networks.

**Table 3:** Factors that dynamically change with exercise.

Dynamic Change	Cytokine	Controls	ME/CFS
↑	Interleukin-2*	√	
	Interleukin-4*	√	√
	Interleukin-5*	√	√
	Interleukin-12p40*	√	
	Interleukin-15*	√	√
	Interleukin-17F^†^	√	
	Leukemia inhibitory factor^†^	√	
	CXCL 10		√
	Interferon-β^‡^	√	√
	Tumor necrosis factor-α^†^	√	
	Nerve growth factor	√	√
	Vascular endothelial growth factor	√	√
	Granulocyte colony stimulating factor^§^	√	√
	Granulocyte-macrophage colony-stimulating factor*^§^	√	
	Stem cell factor^§^	√	√
**↓**	Interleukin-8^†^		√
	CXCL1	√	√
	CCL3	√	√
	CCL4		√
	Tumor necrosis factor-related apoptosis-inducing ligand^†^	√	
	Tumor necrosis factor-β		√
	Intercellular adhesion molecule 1		√

Cytokines are presented by categories as interleukin, chemokines, interferon, growth factors and stimulating factors. *Adaptive immunity. ^†^Pro-Inflammatory signaling. ^‡^Anti-inflammatory signaling. ^§^Hematopoiesis √ - dynamic change with exercise.

### Discrimination of ME/CFS case status

**Figure 3:**
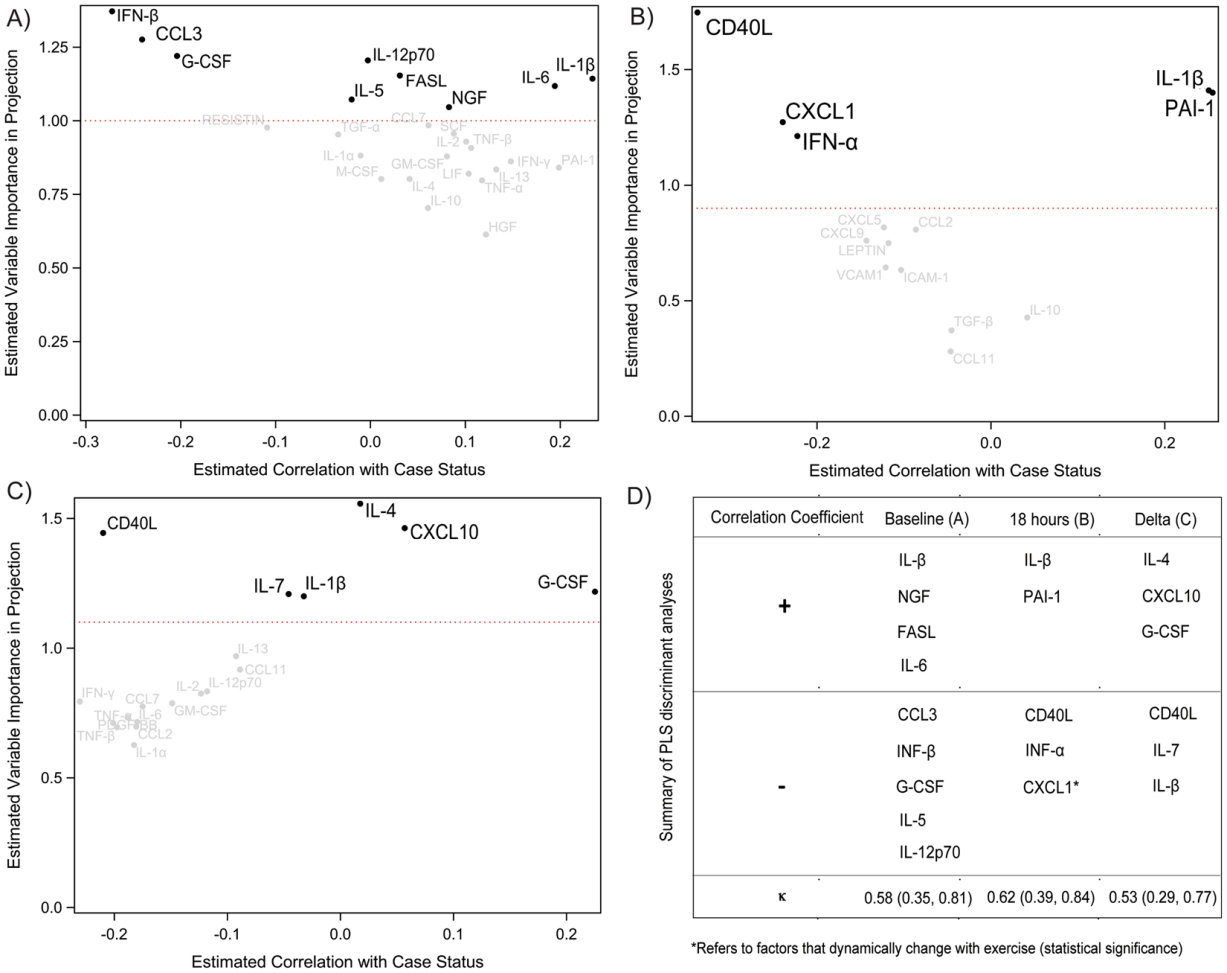
Partial Least Square discriminant analysis to identify factors associated with ME/CFS case status. Kappa (κ) is coefficient of chance-adjusted agreement, here between observed case status and predicted case status from sPLSDA. Cross-validated discriminatory cytokines are those above the horizontal dashed red line. At Baseline (**A**) 18 hours post exercise (**B**) and delta change between A and B (**C**) Summary of sPLSDA with κ coefficients (**D**).

Baseline (at resting), post-exercise (18 hours after exercise) and delta change (between baseline and post exercise) of measured cytokines/growth factors were analyzed using partial sparse least squares analysis to identify factors associated with ME/CFS case status and are summarized in Fig. 3. Cytokines following exercise had nominally better discrimination (greater kappa value) than resting parameters and absolute dynamic change. The factors involved following exercise were related to inflammasome activation (IL-1β), thrombosis (PAI-I), growth factors (CXCL1) immune modulation (INF-α) or co-stimulation activation (CD40L). Among factors, lower CD40L was associated with CFS status. Only one factor was common between baseline and peak exercise (IL-1β, a key cytokines of the inflammasome).

## Discussion

ME/CFS is a major public health problem significantly impairing quality of life. Although efforts have been made to refine the diagnostic criteria and definition of ME/CFS, studying this syndrome remains challenging because of the heterogeneity in presentation, variability in ME/CFS duration and severity and absence of a reliable diagnostic laboratory test or biomarker^10,11^. In this study, we focused on a carefully selected group of ME/CFS patients with significant post-exertional malaise but still able to exercise. Our study has three main findings. First, we have found that exercise can be associated with significant changes in cytokine profile that are still observed 18 hours following symptom-limited exercise. Second, our study suggests that exercise may allow better discrimination of ME/CFS case status than resting values. Third, we have found that cardiac structure at baseline and cardiorespiratory responses following exercise with a one-day protocol do not appear to distinguish cases of ME/CFS from healthy sedentary controls.

Previous studies have analyzed changes in selected cytokines and growth factors profiles but mainly focused on changes following strenuous exercise in athletic participants. Pedersen *et al*. and Toft *et al*. have shown that after a marathon TNF-α and IL-1β levels increase twofold and IL-6 levels increase up to 100-fold but decreases rapidly; this is followed by a marked increase in the concentration of IL-1RA and other anti-inflammatory or regulatory proteins such as IL-8 and IL-10^12–14^. In a recent small study on 10 sedentary individuals, Landers-Ramos *et al*. found that acute exercise (30 minutes of treadmill running at 75% of the subject’s peak VO_2_) increased circulating concentrations of the angiogenic cytokines placental growth factor (PlGF), basic fibroblast growth factor (bFGF) and soluble fms-like tyrosine kinase-1 (sFlt-1), as well as IL-6 and IL-8 in sedentary young men^15^. In addition to the previous angiogenic growth factors, changes in VEGF have not been as extensively studied in healthy participants.

In our study we observed changes in cytokine profiling 18 hours post exercise in both healthy controls and patient with ME/CFS. The biological variability demonstrated in our study has significant implications for the field of cytokine profiling. Greater attention to recent bouts of exercise or activity level should be given as these variables may have implication for data interpretation.

An emerging hypothesis regarding the cause of ME/CFS is immune dysregulation, thought to be reflected in up-regulated pro-inflammatory cytokines leading to the symptoms that are characteristic of this illness. As highlighted by Hornig *et al*. in a large multicenter study (n = 298), cytokine expression in ME/CFS may vary according to the duration of symptoms, stratified in the study to 3 years; expression of cytokines could also vary depending on severity of symptoms. In their study CD40L (a protein of the TNF-receptor superfamily) and platelet-derived growth factor (a growth factor the regulates cell growth and division) were reduced in short duration disease subjects when compared to controls^8^. In contrast Nakamura *et al*. did not observe dynamic change in cytokine profile after exercise or sleep deprivation in a cohort of 26 females with ME/CFS. However, the levels of IL-1β, a cytokine that is part of the inflammasome complex which is often activated in response to metabolic or infectious stress, were higher at both baseline and during exercise in patients with ME/CFS^16^. A further case control study including 24 patients with ME/CFS undertaken by Clark *et al*. was unable to identify meaningful changes in select cytokines post a bout of exercise^17^.

Our study builds on these previous findings adding in terms of originality through the use of a comprehensive immune and growth factor panel (51-plex), a larger cohort of sedentary individuals (in comparison to previous studies) and analysis of persistent changes in circulating factors at 18 hours post exercise. By matching ME/CFS cases and healthy controls samples from day 1 and 2 on the same plate, we minimized the effect of inter-plate variability, in turn providing greater possibility of detecting significant dynamic changes within both cohorts. We found that acute exercise influenced several pathways including inflammatory, growth factors, stem cell factors and vascular factors, some of which persisted up to 18 hours. Consistent with previous exercise studies, elevation of TNF-α post exercise was seen in our sedentary controls, however, no change in IL-6, likely explained by its rapid decrease post exercise and potentially the lower signal to noise ratio of IL-6 on the 51-plex assay. There was also an increase in selected pro-inflammatory cytokines such as IL-2, IL-12p40 and TNF-α in our control group. We applied a network estimation algorithm which is useful for retaining connections between cytokines that are biological significant and removing connections that are statistical noise. Using this method, we found factors with exercise were richly connected, particularly IL-4, which is known to play a key role in immune regulation, specifically Th2 cells. The direct relationship between cytokines however, differed between case and controls networks supportive of a distinct cytokine inflammatory signature in ME/CFS.

Compared to resting cytokine profiles, our study highlights that post-exercise profiling could have greater value in discriminating case status than resting parameters. Among cytokines and growth or vascular factors identified in our discriminatory analyses, CD40L appeared to strongly contribute to discrimination, with negative correlation both 18 hours post exercise and through its change from baseline. This is consistent with previous findings indicating that a failure to reduced levels of CD40L post exercise is associated with increased symptom flare post a bout of moderate exercise^18^. The association with CD40L was also found in a recent larger study, at rest by our group (with no overlap of patients) with a trend for lower levels of CD40L compared to controls across the spectrum of disease severity^19^.

CXCL1, like CD40L contributed strongly to multivariable discrimination of cases and controls post exercise. Unlikely CD40L, in univariate analysis separately by cytokine, CXCL1 decreased with exercise in controls and cases. CXCL1 also demonstrated high relative centrality within the network participants with ME/CFS. Interestingly, increased CXCL1 production by neutrophils has been seen in patients with fibromyalgia, however, considering the small numbers within the studies, these findings should be considered exploratory and further investigation is required to define their clinical implications^20^.

Further supportive of an immune mediate pathway in ME/CFS, we found CXCL10 played a central role in the cytokine network and contributed to case discrimination when combine with delta change in IL-4, G-CSF, IL-1β, IL-7 and CD40L. Recently CXCL10 has been shown to play a role in autoimmune disease, in particular type 1 diabetes and inflammatory bowel disease, through the augmentation of the Th1 autoimmune response^21,22^. Further studies will be required to define its contribution to ME/CFS.

Several small studies initially suggested differences in cardiac size between ME/CFS and healthy controls^3,4^. Using patients well matched for level of activity, we were unable to find significant differences in cardiac structure or function in our ME/CFS cohort with regard to fitness independent measures of ventricular remodeling such as mass to volume ratio or scaled ventricular dimension. Similarly, we were unable to identify differences in vascular stiffness using central aortic pulse wave velocity or significant differences in endothelial function, peak VO_2_ or ventilatory efficiency. The fact that no difference was detected between CPET parameters between both groups despite marked difference in MFI-20 scores highlights the difference between reported symptoms such as fatigue or dyspnea and exercise performance measured by peak VO_2_^23^. This underscores the importance of not using these two concepts interchangeably.

Regarding exercise protocol our study used a single submaximal exercise protocol with repeat blood draw 18 hours post exercise to correlate with the onset of post exercise malaise, optimize processing of serum samples and to determine whether cytokine profiling could better discriminate than CPET parameters on day 1. However, in 2007 a seminal study by Snell *et al*. demonstrated the value in using a two day CPET protocol, through diminished CPET performed a day after the first^24^. Contemporary studies have confirmed these findings and suggested the use of a two-day CPET challenge protocol when assessing patients with ME/CFS in particularly those with post exercise malaise^25–27^.

Our study has several limitations. First, although our patients were carefully selected and matched with sedentary controls, the sample size is small with a small but statistically significant difference in BMI between groups. There was however, no difference in the number of participants, overweight or obese by this classification. In an effort to avoid false discovery rates, we also conducted careful adjustment of multiple measures. The fact that exercise was able to reveal similar factors of the larger resting study of Hornig *et al*. increased confidence in our findings. Despite no exercise validation cohort, the fact that several factors discussed emerged in both patients with ME/CFS and sedentary controls also brings more confidence in the results. Luminex assays are also known not to have a good signal to noise ratio for IL-6, however, our findings appear consistent with contemporary studies. Finally, we selected a sub-group of patients with ME/CFS, with severe post exercise fatigue to ensure a more specific phenotype.

In conclusion, our study suggests that exercise may be useful to profile key biological difference in ME/CFS and sedentary controls. We also highlight the importance to account for exercise when profiling disease states or syndromes. Replicating the findings and investigating profiling using a two-day protocol will be important steps for future research.

## Materials and Methods

This study was approved by the Stanford Institutional Review Board (IRB) with all protocols conducted in accordance with relevant guidelines and regulations. Informed consent was obtained from all patients recruited. The recruitment of patients was performed using the Stanford Translational Research Integrated Database Environment (STRIDE), which is a secure database that summarized key characteristics of patients with ME/CFS followed as part of the Stanford University ME/CFS Initiative. We selected patients older than 18 years old with symptoms lasting more than one year with a component of severe post exertional malaise and fatigue. When designing the study we used the 1994 Centers for Disease Control (CDC)/Fukuda international diagnostic criteria for ME/CFS, but required participants to have post exertional malaise. Therefore, in labeling our patients this refers to the revised international consensus criteria from 2011^28^. Patients with ME/CFS were matched to healthy sedentary volunteers according to age, sex, and race. Sedentary state was defined by an activity of up to six Metabolic Equivalents of Task (METS) less than once a week and of less than one-hour duration, or by an activity of less than 4 METS less than three times a week and less than one hour each time as per the International Physical Activity Questionnaire (IPAQ)^29^. Subjects with a history of active inflammatory disease, acute infection within 30 days prior to the study day, systemic hypertension, pulmonary disease, obstructive sleep apnea (OSA), diabetes mellitus, dyslipidemia, obesity (defined by a body mass index (BMI) >30 kg/m^2^), coronary artery disease, chest pain of unknown origin, erectile dysfunction, anemia with hemoglobin level below 110 g/L, smoking, hypothyroidism, fibromyalgia, psychiatric disorders and malignancy were excluded. In addition, subclinical cardiovascular disease was excluded using echocardiography and carotid and femoral artery ultrasound examination. Patients with left ventricular ejection fraction <50%, moderate diastolic dysfunction or carotid or femoral plaque greater than 20% diameter stenosis were excluded^30,31^.

### Questionnaires

Participants were administered three questionnaires; a health screening questionnaire, a standardized fatigue questionnaire (MFI-20) and IPAQ 2005. The MFI-20 questionnaire is a recommended tool to evaluate fatigue in patients with ME/CFS^32,33^. It is a self-reported questionnaire resulting in a single score where higher scores indicate greater fatigue and disease severity. The IPAQ is a standardized questionnaire to evaluate physical activity and was used to ensure that controls being recruited were sedentary^29^.

### Baseline Screening and Exercise protocol

Screening for subclinical atherosclerosis or ventricular dysfunction was performed using vascular ultrasound of the carotid and femoral artery with a 9.0 MHz Philips linear array probe and iE33 xMATRIX echocardiography System manufactured by Philips® (Andover, MA, USA)^31^. Vascular stiffness was assessed using central pulse wave velocity and ultrasound methodology, endothelial function was assessed using ENDOPAT ® both at rest and after exercise^34^.

The exercise protocol was performed using an upright ergocycle and an individualized one-day ramp protocol with increments of 15 to 25 Watts per 90 seconds^35^. All participants underwent symptom limited exercise. Ventilatory expired gas analysis was completed using the Shape Medical system^36^. Minute ventilation (VE), oxygen uptake (VO_2_), carbon dioxide production (VCO_2_) were acquired breath by breath and averaged over 10 second intervals. VE and VCO_2_ responses throughout exercise were used to calculate the VE/VCO_2_ slope via least squares linear regression (y = mx + b, m = slope) as validated through previous studies^37,38^.

### Sample Preparation, Baseline Metabolic Profiles and Cytokine assay

All samples collected were drawn fasting in the morning so they could be processed immediately (within 60 minutes) and brought to a −80 freeze; none of the samples underwent unfreezing. All samples were analyzed simultaneously within 3 month of completion the study with laboratory staff blinded to group membership.

The assays were performed on serum samples in the Human Immune Monitoring Center (HIMC) at Stanford University. Baseline levels for the metabolic panel, lipid panel, thyroid stimulating hormone and high-sensitivity C reactive protein were assessed. To measure a panel of cytokine and growth factors we used a 51-plex Luminex bead kit (Affymetrix, Santa Clara, CA) (Table 4). Each sample was measured in duplicate. Plates were read using a Luminex LabMap200 instrument with a lower bound of 100 beads per sample per measured cytokine^8^. The Luminex LabMap200 outputs the fluorescence intensity of each bead measured for a given cytokine in a sample. For each well, we used the median fluorescence intensity (MFI) of all beads measured for a given cytokine and averaged the MFI of the two replicates. By design, ME/CFS cases and healthy controls samples were age, sex and race matched on each plate as well as the samples from day 1 and day 2, to minimize confounding of plate artifacts with clinical comparisons of interest. Three plates were used for the assays and the coefficient of variation between assays for all biomarkers was <10% for the majority of cytokines.

**Table 4:** Cytokines assessed in the multiplex assay.

Class	Cytokine
Growth factors	FGF-β, HGF, NGF, PDGF-BB, TGFα, TGF-β1, VEGF
Colony stimulating factors and stem cell factors	G-CSF, GM-CSF, M-CSF, SCF
Interleukins	IL-1α, IL-1β, IL-1RA, IL-2, IL-4, IL-5, IL-6, IL-7, IL-8, IL-10, IL12p40, IL12p70, IL-13, IL-15, IL-17, IL-17F, IL-18 and LIF
Chemokines	CCL2 (MCP-1), CCL3 (MIP-1α), CCL4 (MIP-1β), CCL5 (RANTES) CCL7 (MCP-3), CXCL1 (Gro-α), CXCL5 (ENA78), CXCL9 (MIG), CXCL10 (IP-10), CCL11 (Eotaxin)
Interferons	INF-α, INF-β, INF-ϒ
Adhesion Molecules	ICAM-1, VCAM-1
Other factors	CD40L, FASL, Leptin, PAI-1, Resistin, TNF-α, TNF-β, TRAIL

CD40L, CD40 ligand; CCL, Chemokine (C-C motif) ligand; CXCL, chemokine (C-X-C motif) ligand; ENA-78, epithelial neutrophil activating peptide-78; FASL, Fas Ligand FGF-β, fibroblast growth factor-β; G-CSF, granulocyte colony stimulating factor; GM-CSF, granulocyte-macrophage colony-stimulating factor; Gro-α, growth-regulated α protein; HGF, hepatocyte growth factor; ICAM-1, intercellular adhesion molecule 1; IL, interleukin; IL-RA, interleukin-receptor antagonist; INF, interferon; **IP-10, interferon gamma-induced protein**, LIF, leukemia inhibitory factor; MCP, monocyte chemotactic protein; M-CSF, macrophage colony-stimulating factor; **MIG**, monokine induced by gamma interferon; MIP, macrophage inflammatory protein; NGF, nerve growth factor; PDGF-BB, platelet-derived growth factor-BB; PIGF-1, placenta growth factor-1; RANTES, regulated upon activation, normal T cell expressed and secreted; SCF, stem cell factor; **1α;** TGF, transforming growth factor; TNF, tumor necrosis factor; TRAIL, tumor necrosis factor-related apoptosis-inducing ligand; VCAM-1, vascular cell adhesion molecule 1; VEGF, vascular endothelial growth factor.

We pre-processed MFI data for all subsequent statistical analyses. First, each cytokine’s MFI values were averaged over duplicate wells and base-2 logarithmically transformed, yielding log_2_(avg MFI). Next, we subtracted the mean internal control log_2_(avg MFI) value by plate from the corresponding log_2_(avg MFI) values for any cytokine for which internal control log_2_(avg MFI) values demonstrated a statistically significant (p < 0.05) linear trend across plates. Then we detrended all resultant log_2_(avg MFI) values for nonspecific binding by regressing these values on log_2_(avg MFI) for CHEX4 beads (Montoya *et al*.^19^). To complete this transformation process, separately for each cytokine, CHEX4-detrended log_2_(avg MFI) values were centered and scaled as (x − m)/s for sample mean m and standard deviation s.

### Statistical Methods and Identification of Biomarkers

Statistical analysis was completed with assistance from the Stanford Immune Institute Bioinformatics Core. Groups’ means were compared at baseline^39^. Initial data preparation consisted of calculating average median fluorescence intensity (MFI) over each pair of duplicate wells per person per visit and taking logarithm (base 2) of each resultant average (hereafter “log MFI”). Linear mixed-coefficient modeling (LMM) with a random intercept per participant was used to test for change in MFI between baseline and 24 hours^40^. False discovery rate was controlled across all 51 cytokines using Benjamani *et al*.^41^ per Kim and van de Weil^42^. A mutual information network was estimated for cytokines demonstrating change^43^. We identified possible predictors of case status using sparse partial least squares discriminant analysis (sPLSDA) via bagged training/validation^44^. LMM and sPLSDA were adjusted for age, gender, BMI, and nonspecific binding^19^. The kappa coefficient (κ) was used to assess chance-adjusted degree of agreement between observed case status versus predicted case status with a κ > 0.80 having excellent discrimination, κ between 0.61 to 0.80, substantial discrimination, κ between 0.41 to 0.60, moderate discrimination, κ between 0.21 to 0.40, fair discrimination and κ < 0.20, poor (slight) discrimination^45^. Partial Spearman’s rank correlation coefficients were estimated between each cytokine and each of the endothelial function and cardiopulmonary exercise testing (CPX) variables after taking into account non-specific binding, age, sex, BMI and multiple comparisons. Statistical analysis was completed using MedCalc v15.8 (MedCalc Software, Ostend, Belgium), SAS® v.9.4 (SAS® Institute, Cary, North Carolina, USA), R v.3.2.2 through v.3.3.2 (https://www.R-project.org), and Mathematica v.11 (Wolfram Research, Champaign, Illinois, USA).

### Data availability

The datasets generated during and/or analyzed during the current study are available from the corresponding author on reasonable request.

## References

[1] Prins JB, van der Meer JW, Bleijenberg G (2006). Chronic fatigue syndrome. Lancet.

[2] Committee on the Diagnostic Criteria for Myalgic Encephalomyelitis/Chronic Fatigue, S., Board on the Health of Select, P. & Institute of, M. In *Beyond Myalgic Encephalomyelitis/Chronic Fatigue Syndrome: Redefining an Illness**www.nap.edu/download/19012* (2015)

[3] Hollingsworth KG, Hodgson T, Macgowan GA, Blamire AM, Newton JL (2012). Impaired cardiac function in chronic fatigue syndrome measured using magnetic resonance cardiac tagging. J Intern Med.

[4] Miwa K, Fujita M (2009). Cardiovascular Dysfunction with Low Cardiac Output Due to a Small Heart in Patients with Chronic Fatigue Syndrome. Internal Medicine.

[5] Lorusso L (2009). Immunological aspects of chronic fatigue syndrome. Autoimmunity reviews.

[6] Raison CL, Lin JM, Reeves WC (2009). Association of peripheral inflammatory markers with chronic fatigue in a population-based sample. Brain Behav Immun.

[7] Blundell, S., Ray, K. K., Buckland, M. & White, P. D. Chronic fatigue syndrome and circulating cytokines: A systematic review. *Brain Behav Immun*, 10.1016/j.bbi.2015.07.004 (2015). 10.1016/j.bbi.2015.07.004 26148446

[8] Hornig M (2015). Distinct plasma immune signatures in ME/CFS are present early in the course of illness. Science Advances,.

[9] Hornig M (2017). Immune network analysis of cerebrospinal fluid in myalgic encephalomyelitis/chronic fatigue syndrome with atypical and classical presentations. Transl Psychiatry.

[10] Clayton EW (2015). Beyond myalgic encephalomyelitis/chronic fatigue syndrome: an IOM report on redefining an illness. JAMA.

[11] Komaroff AL (2015). Myalgic Encephalomyelitis/Chronic Fatigue Syndrome: A Real Illness. Ann Intern Med.

[12] Ostrowski K (1998). A trauma-like elevation of plasma cytokines in humans in response to treadmill running. Journal of Physiology.

[13] Ostrowski K, Rohde T, Asp S, Schjerling P, Pedersen BK (1999). Pro- and anti-inflammatory cytokine balance in strenuous exercise in humans. Journal of Physiology.

[14] Pedersen BK, Steensberg A, Schjerling P (2001). Exercise and interleukin-6. Curr Opin Hematol.

[15] Landers-Ramos RQ, Jenkins NT, Spangenburg EE, Hagberg JM, Prior SJ (2014). Circulating angiogenic and inflammatory cytokine responses to acute aerobic exercise in trained and sedentary young men. Eur J Appl Physiol.

[16] Nakamura T (2013). Exercise and Sleep Deprivation Do Not Change Cytokine Expression Levels in Patients with Chronic Fatigue Syndrome. Clin Vaccine Immunol.

[17] Clark LV (2017). Cytokine responses to exercise and activity in patients with chronic fatigue syndrome: case-control study. Clinical and experimental immunology.

[18] White AT (2010). Severity of symptom flare after moderate exercise is linked to cytokine activity in chronic fatigue syndrome. Psychophysiology.

[19] Montoya, J. *et al*. A Novel Cytokine Signature Associated with Disease Severity in Chronic Fatigue Syndrome Patients. *PNAS* (2017). 10.1073/pnas.1710519114 PMC5576836 28760971

[20] Garcia JJ, Carvajal-Gil J, Guerrero-Bonmatty R (2016). Altered release of chemokines by phagocytes from fibromyalgia patients: a pilot study. Innate Immun.

[21] Singh UP (2016). Chemokine and cytokine levels in inflammatory bowel disease patients. Cytokine.

[22] Antonelli A, Ferrari SM, Corrado A, Ferrannini E, Fallahi P (2014). CXCR3, CXCL10 and type 1 diabetes. Cytokine Growth Factor Rev.

[23] Myers J (2006). Association of functional and health status measures in heart failure. J Card Fail.

[24] Vanness JM, Snell CR, Stevens SR (2007). Diminished Cardiopulmonary Capacity During Post-Exertional Malaise. Journal of Chronic Fatigue Syndrome.

[25] Snell CR, Stevens SR, Davenport TE, Van Ness JM (2013). Discriminative validity of metabolic and workload measurements for identifying people with chronic fatigue syndrome. Physical therapy.

[26] Keller BA, Pryor JL, Giloteaux L (2014). Inability of myalgic encephalomyelitis/chronic fatigue syndrome patients to reproduce VO(2)peak indicates functional impairment. Journal of translational medicine.

[27] Nijs J (2014). Altered immune response to exercise in patients with chronic fatigue syndrome/myalgic encephalomyelitis: a systematic literature review. Exercise immunology review.

[28] Carruthers BM (2011). Myalgic encephalomyelitis: International Consensus Criteria. J Intern Med.

[29] Craig CL (2003). International physical activity questionnaire: 12-country reliability and validity. Med Sci Sports Exerc.

[30] Lang RM (2015). Recommendations for cardiac chamber quantification by echocardiography in adults: an update from the American Society of Echocardiography and the European Association of Cardiovascular Imaging. J Am Soc Echocardiogr.

[31] Gerhard-Herman M (2006). Guidelines for noninvasive vascular laboratory testing: a report from the American Society of Echocardiography and the Society of Vascular Medicine and Biology. J Am Soc Echocardiogr.

[32] Lin JM (2009). Further validation of the Multidimensional Fatigue Inventory in a US adult population sample. Popul Health Metr.

[33] Williams DA, Arnold LM (2011). Measures of fibromyalgia: Fibromyalgia Impact Questionnaire (FIQ), Brief Pain Inventory (BPI), Multidimensional Fatigue Inventory (MFI-20), Medical Outcomes Study (MOS) Sleep Scale, and Multiple Ability Self-Report Questionnaire (MASQ). Arthritis Care Res (Hoboken).

[34] Flammer AJ (2012). The assessment of endothelial function: from research into clinical practice. Circulation.

[35] Shimizu M (1991). The ventilatory threshold: method, protocol, and evaluator agreement. Am Heart J.

[36] Miller AD (2010). Validation of a Simplified, Portable Cardiopulmonary Gas Exchange System for Submaximal Exercise Testing. The Open Sports Medicine Journal.

[37] Arena R, Myers J, Aslam S, Varughese E, Peberdy M (2003). Technical considerations related to the minute ventilation/carbon dioxide output slope in patients with heart failure. Chest.

[38] Arena R (2009). Determining the preferred percent-predicted equation for peak oxygen consumption in patients with heart failure. Circ Heart Fail.

[39] Golan, A., Judge, G. & Miller, D. Maximum entropy econometrics: robust estimation with limited data. (John Wiley & Sons, Inc., 1996).

[40] McCulloch, C. & Searle, S. Generalized, linear and mixed models. (John Wiley & Sons, Inc., 2001).

[41] Benjamini Y, Krieger AM, Yekutieli D (2006). Adaptive linear step-up procedures that control the false discovery rate. Biometrika.

[42] Kim KI, van de Wiel MA (2008). Effects of dependence in high-dimensional multiple testing problems. BMC Bioinformatics.

[43] Meyer PE, Lafitte F, Bontempi G (2008). minet: A R/Bioconductor package for inferring large transcriptional networks using mutual information. BMC Bioinformatics.

[44] Le Cao K-A, Boitard S, Besse P (2011). Sparse PLS discriminant analysis: biologically relevant feature selection and graphical displays for multiclass problems. BMC Bioinformatics.

[45] Landis Fau - Koch, G. G. Jr. & Koch, G. G. The measurement of observer agreement for categorical data (0006-341X (Print)). 843571

